# Childhood cerebral adrenoleukodystrophy (CCALD) in France: epidemiology, natural history, and burden of disease - A population-based study

**DOI:** 10.1186/s13023-023-02843-x

**Published:** 2023-08-10

**Authors:** Caroline Sevin, Samira Hatteb, Aurore Clément, Fabrizia Bignami, Louis Chillotti, Françoise Bugnard, Stève Bénard, Odile Boespflug-Tanguy

**Affiliations:** 1 Center of Reference for Leukodystrophies, Bicêtre Hospital - APHP, Le Kremlin Bicêtre, France; 2 Blueblird Bio, Somerville, MA US; 3 Stève consultants, Oullins, France; 4 Center of Reference for Leukodystrophies, Robert Debré Hospital - APHP, Paris, France

**Keywords:** Adrenoleukodystrophy, CCALD, Epidemiology, Claims, Registry, HSCT, SNDS, Care management, Neurodegenerative diseases, Health cost

## Abstract

**Background:**

X-linked adrenoleukodystrophy (ALD) is a rare metabolic and neurodegenerative disorder belonging to the group of leukodystrophies, with an estimated incidence around 1:25 000 newborns worldwide, mostly among men. Childhood Cerebral ALD (CCALD) is the most severe form with a poor prognosis if not properly treated during the first years of life. Currently, only allogeneic hematopoietic stem cell transplantation (allo-HSCT) is widely available for CCALD treatment. To date, there is a lack of data regarding CCALD epidemiology, natural history, and current management in France. This knowledge is crucial for the development of new therapies such as gene therapies. In this context, the French National Health Data System (SNDS) is a particularly indicated database to collect information meeting these needs. A non-interventional, national, real-life, retrospective study was performed using secondary data from the national ALD registry (LEUKOFRANCE) and SNDS. CCALD patients detected between 2009 and 2018 and successfully matched between LEUKOFRANCE and SNDS were included in this study. Index date was defined as the first CCALD event detected during study period. Subgroups of patients with sufficient follow-up (6 months) and history (1 year) available around index date were analyzed to assess CCALD burden and natural history.

**Results:**

52 patients were included into the matched cohort. Median annual incidence of CCALD was estimated at 4 patients. Median age at CCALD diagnosis was 7.0 years. Among patients without allo-HSCT, five-year overall survival was 66.6%, with 93.3% of them presenting at least one CCALD symptom and 62.1% presenting a least one major functional disability (MFD). Among patients with allo-HSCT, five-year overall survival was 94.4%, with only 11.1% of patients presenting CCALD symptoms, and 16.7% of presenting a MFD. Mean annualized costs were almost twice as important among patients without allo-HSCT, with 49,211€, 23,117€, respectively. Costs were almost exclusively represented by hospitalizations.

**Conclusions:**

To the best of our knowledge, this is the most up to date study analyzing CCALD epidemiology, clinical and economic burden in France. The necessity of a precocious management with HSCT highlight the potential benefits of including an expanded screening program among newborns, coupled with family screenings when a mutation is detected.

## Background and rationale

X-linked adrenoleukodystrophy (ALD) is a rare metabolic and neurodegenerative disorder belonging to the group of leukodystrophies. It results in the production of a dysfunctional adrenoleukodystrophy protein (ALDP), leading to an accumulation of very long chain fatty acids in plasma and tissues, causing axonal damage, cerebral demyelination, and adrenal insufficiency. ALD overall incidence rate, including hemizygotes and heterozygotes who are frequently symptomatic, is estimated between 1:15 000 and 1:33 000 newborns worldwide [1–4]. Most frequently observed clinical manifestations are in line with three main ALD phenotypes. The most frequent and mildest form is an isolated adrenal insufficiency, or Addison disease, observed among around 50% of X-ALD patients, leading to hypotension, hypoglycemia, fatigue, or joint pain. The second main form of X-ALD is Adrenomyeloneuropathy (AMN), an adult-onset slowly progressive myelopathy and peripheral neuropathy, that begins between the ages of 20 and 40 with full penetrance beyond age 60 in men and may also affect heterozygous females. Finally, the most rapidly progressive and devastating form of ALD form of X-ALD is Childhood Cerebral ALD (CCALD), occurring within the first 10 years of life. CCALD has an insidious onset and leads to important neurologic disorders in addition to Addison disease. The main neurologic manifestations occurring during disease progression include hyperactive behavior, auditory and visual impairment, hemiparesis, cerebellar ataxia or spastic tetraparesia which can become highly debilitating within weeks or months. This suddenly progressive disease form is observed for around 40% of boys and adolescents affected with CCALD, with a very low life expectancy without treatment. Less invasive phenotypes will have the same evolution pattern with an onset around 10 to 15 years of age [5–7].

Apart from family screening, the majority of patients are diagnosed when they already have symptoms, a time when the disease is too advanced to offer a curative treatment. Aside from the treatment of the Addison disease, which relies on adrenal hormone supplementation, patients will benefit from symptomatic and palliative treatment (pain, spasticity, nutritional disorders, swallowing disorders, orthopedic complications, physiotherapy) to allow improving their quality of life. Allogeneic hematopoietic stem cell transplantation (allo-HSCT) is the only curative treatment approved in France, which has been shown to have a beneficial effect on clinical indices of disease and long-term survival [8]. However, it must be performed at the earliest stage of cerebral demyelination process to be effective [5, 6]. An international consensus confers optimal eligibility for HSCT by modern standards: neurological functional score (NFS) ≤ 1, demyelinating score (Loes score) of 0.5 to ≤ 9, and gadolinium enhancement in cerebral imaging [9–11]. For more severe patients, allo-HSCT is not recommended, as allo-HSCT will need weeks to months to be effective and will not tackle the rapid disease evolution, leading to irreversible brain damage. Only a limited number of patients will actually present all required conditions to benefit from an allo-HSCT, which remains associated to a significant mortality risk, reaching up to 15–25% in children during the first year post HSCT, due to graft failure, graft versus host disease (GVHD) and opportunistic infections [12]. Recently, ex vivo gene therapy has been developed for CCALD and evaluated as an interesting alternative to allograft. Clinical trials performed in CCALD children demonstrate that this treatment, if performed in the same indications than allo-HSCT, provides similar efficacy [13].

To date, there is a lack of data regarding CCALD epidemiology, natural history, and current management in France. This knowledge is crucial for the development of new therapies. In addition, from health policies perspective, there is a need of comprehensive and recent data on CCALD impact on public health, based on healthcare resource use and related costs from Health Insurance and Collective perspectives. In this context, the French National Health Data System (*Système National des données de Santé*, SNDS) is a particularly indicated database to collect information meeting these needs.

## Objectives

In order to answer these questions, four objectives were set up.

The main objective was (objective A) to determine the **number of patients** with CCALD included in a matched cohort based on SNDS data and French registry of Leukodystrophies (LEUKOFRANCE), between 2009 and 2018, overall and per year, and to describe their characteristics.

Secondary objectives are listed as follows:

- to describe CCALD **natural history**, and notably (i) the delay between ALD diagnosis and the occurrence of the CCALD phenotype, (ii) the nature and frequency of morbidity CCALD-related events and (iii) the overall survival and most common causes of death (objective B);
- to assess the CCALD **care management**, including medical procedures and therapies, and to describe the disease evolution depending on the therapeutic management, notably according to HSCT status (objective C);
- to assess **health care resource use** of CCALD patients and **related costs**, form the Health Insurance and the collective perspective (objective D).

## Methods

### Study design

The study was a non-interventional, national, real-life, retrospective study with secondary use of two sources of existing data. The primary source was the national leukodystrophies registry, called LEUKOFRANCE registry, which includes patients referred to the French reference centers for leukodystrophies (LEUKOFRANCE). The CCALD patients extracted from this registry were probabilistically linked to the medico-administrative database of the French Health Insurance (French System of Health Data - Système National des Données de Santé, SNDS) as a secondary source. Patients with a CCALD-related event (diagnosis and/or treatment) recorded between January 1st 2009 and June 31st 2018 and successfully matched between LEUKOFRANCE registry and SNDS data were included in this study. Index date was defined as the date of first CCALD diagnosis, allowing to distinguish prevalent patients with an index date captured using LEUKOFRANCE registry data prior to 2009, and incident patients (i.e., with an index date during study period).

A first subgroup of patients was followed-up for a minimum of 6 months until December 31st, 2018 (corresponding to the end of study period) or death, whichever occurs first, to assess clinical and economic burden of the disease (objectives C and D). Among them, those having at least 1 year of historical data before index date were extracted to assess CCALD natural history (objective B).

### Data sources

#### LEUKOFRANCE registry

LEUKOFRANCE registry belongs to the Reference Centre for Leukodystrophies and Rare Leukoencephalopathies (LEUKOFRANCE). In 2013 (corresponding to the middle of the study period), almost 2,000 families were part of LEUKOFRANCE registry, gathering phenotypic, imaging, molecular, clinical resources, and data, as well as almost 30,000 biological samples. When considering historical data retrospectively included, LEUKOFRANCE registry allows access to more than 30 years of data, with first record available dating back to 1988. Among data of interest, sociodemographic characteristics, date of diagnosis, symptom onset, imagery, and biology results, as well as ALD related events and treatments were analyzed.

#### SNDS

The national health data system, SNDS (*Système National des Données de Santé*) is managed by the national health insurance since its implementation in 2017 [14]. It gathers and allows the linkage of all reimbursed healthcare consumptions from inpatient and outpatient databases. It covers every subjects affiliated to one of the compulsory health insurance plans, representing nearly 99% of French residents [15, 16]. Each data is returned in an individualized way using a unique pseudonymized identification number called NIR. Data collected include administrative information on the beneficiaries, all reimbursed ambulatory health care services (e.g. health professionals, date, nature and number of care performed, biology acts, reimbursable medical devices, dispensed drugs – recorded with their respective classification system…) as well as those performed in healthcare facilities (type of facility, dates and modes and entry and discharge, diagnoses, procedures…) [17, 18].

### Study population

Study population included all CCALD patients successfully linked from LEUKOFRANCE registry and SNDS and will be referred as LEUKOFRANCE-CCALD cohort.

SNDS cohort included male beneficiaries, with at least one diagnosis code potentially related to ALD, using the international classification of diseases – 10 revision (ICD-10) codes, and aged less than 25 years old at the first ICD10 coded recorded. ICD-10 codes used for ALD detection included E71.3, G13, G31.8, G31.9, G37.8, G37.9, G53.8, G64, G94, G95, and G99 [19]. Exhaustive list and code detail are available in supplementary appendix.

LEUKOFRANCE cohort included ALD patients alive on January 1st, 2009, with a diagnosis of CCALD before their 18th anniversary.

The creation of the LEUKOFRANCE-CCALD cohort was based on a 2-stepped probabilistic linkage. The first step was to detect patients from SNDS with at least one stay at an ALD reference center (Saint Vincent de Paul hospital and Bicêtre hospitals, *Assistance Publique des Hôpitaux de Paris*), as every CCALD patients from LEUKOFRANCE registry went to these hospitals. The second step was the probabilistic linkage *per se* using variables available in both databases. Linkage algorithm used gender, date of birth, region of residence, date of diagnosis, date of specific biology tests (cortisol, VLCFA…) date of brain imagery, date of HSCT among others. Based on literature data and previous experience, a linkage rate of at least 90% was expected [20–22]. A medical review was performed for LEUKOFRANCE patients with multiple matches.

As HSCT remains one of the most effective therapies for CCALD and represents a drastic change in disease management and symptomatology, patients were analyzed according to their HSCT status, between patients who did or did not undergo HSCT. Patients were also analyzed according to the way ALD diagnosis was performed. Screened cases include patients who underwent a precocious targeted test for ALD, mostly due to family history or isolated adrenal insufficiency. Index cases include patients who were fortuitously diagnosed or diagnosed when first symptoms occurred.

### Study variables

Variables of interest included patient’s sociodemographic characteristics at index date (age, gender, area of residency…), disease natural history (type and date of CCALD symptoms, Major Functional Disabilities [MFD – blindness, communication loss, enteral nutrition, incontinence, movement loss and wheelchair use], HSCT, death…), disease management (laboratory and imaging, CCALD treatments), healthcare resource use and their related costs (inpatient stays, home hospitalization, reimbursed medical and paramedical cares, outpatient visits, treatments, medical devices, transportations…).

Classifications and codes used for variable detection gather the **ICD10** classification for diseases and diagnoses, the ***Classification Commune des Actes Médicaux*** (CCAM) for medical procedures [23], the ***Nomenclature des actes de biologie médicale*** (NABM) for outpatient laboratory tests [24], the ***Liste des Produits et Prestations*** (LPP) for reimbursed medical devices [25]. Drugs were detected using either the international anatomical, therapeutic and chemical classification (**ATC**), or specific French codes for more detailed information about packaging such as the ***Code Identifiant de Presentation*** (CIP) [26, 27].

The exhaustive list of variables and their availability among both databases, as well as coding algorithms used for their detection, are available in Supplementary appendix.

### Statistical methods

All analyses have been performed using SAS® version 9.4 (SAS Institute Inc. Cary, NC, USA). Quantitative variables were described in terms of mean, standard deviation, median, quartiles and extreme values. Qualitative variables were described in terms of absolute value frequency and percentage for each category by modality, excluding missing data. In accordance with data protection requirements, clinical categories extracted from SNDS data including less than 11 patients may be either grouped with other categories or not described. Cost categories are not affected by this measure. MFD-free and overall survivals were assessed from CCALD diagnosis. For patients presenting MFD before CCALD diagnosis, MFD was considered to occur at CCALD diagnosis. Overall survival and MFD-free survival were estimated using Kaplan-Meier method. MFD-free survival events of interest were defined for patients with HSCT as the first MFD occurrence or a second HSCT or death, whichever occurred first. For patients without HSCT, first MFD occurrence or death were considered, whichever occurs first. Cost analysis was performed based on health care consumptions of interest identified for the study population. Costs per patient for each health care of interest (i.e., direct medical costs) were extracted from the SNDS database. Costs presented for reimbursement and costs reimbursed by the French Health Insurance were extracted from the above database, to perform the economic analysis from the collective perspective and the French Health Insurance perspective, respectively. Costs are reported in Euros (€), year 2018, with costs prior to 2018 being revalued according to a consumer price index published by the national institute for statistics (INSEE) [28].

## Results

### CCALD LEUKOFRANCE-SNDS Cohort (Objective A)

**Fig. 1:**
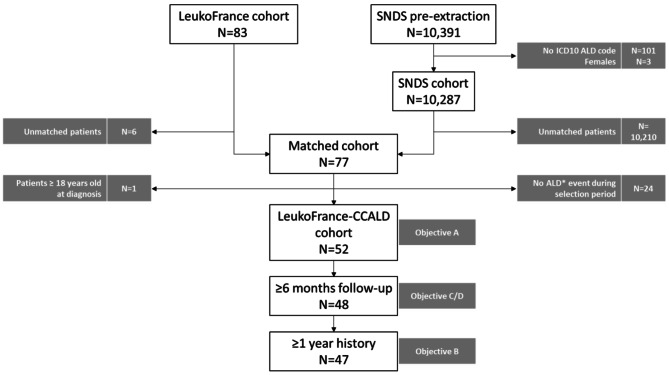
Patient Disposition (N = 52) *ICD-10 ALD codes (E71.3, G13, G31.8, G31.9, G37.8, G37.9, G53.8, G64, G94, G95, G99)* *ALD events: hospitalization or Long Duration Disease record with ICD-10 ALD codes*

Among the 10,391 patients pre-identified in SNDS database, 10,287 (99.0%) were extracted for matching (SNDS cohort), based on validated ICD-10 codes related to ALD. In the other hand, LEUKOFRANCE cohort included 83 (90.2%) patients among the 92 identified within the registry. After the 2-stepped linkage, 77 patients were successfully matched, leading to a linkage rate of 92.8%, with 6 patients unmatched. Finally, 52 patients with at least one CCALD-related event during study period and sufficient data depth were included into the LEUKOFRANCE-CCALD cohort, composing the initial study population (Fig. 1).

Among these patients, mean (SD) follow-up duration after CCALD diagnosis was 89.4 (77.8) months, with 68.1 (42.7) months during study period.

Over study period, median annual incidence of CCALD was estimated at 4 patients, ranging between 2 and 6, leading to 35 incident cases during study period and 17 prevalent ones. Median age at CCALD diagnosis was 7.0 years, ranging from 0.5 (screened case) to 13 years of age, while more than half (51.9%) of patients were diagnosed between 5 and 8 years of age. A majority of patients did not have a family history for CCALD (Table 1).

**Table 1:** Patients’ characteristics

Characteristics		LEUKOFRANCE CCALD cohort (N = 52)
**Age (y)**	median (Q1-Q3)	7.0 (6.0–9.0)
min-max		0.5–13.0
**Age classes (y)**		
< 3 years old	N (%)	2 (3.8)
[3–5[ years old		2 (3.8)
[5–8[ years old		27 (51.9)
[8–12[ years old		17 (32.7)
[12–15[ years old		4 (7.7)
[15–18[ years old		0 (0.0)
**ALD family history**		
yes	N (%)	15 (29.4)
no		36 (70.6)
**Incident cases**		
2009	N	2
2010		3
2011		5
2012		2
2013		4
2014		2
2015		6
2016		3
2017		4
2018		4
10-year study period	N (%)	35 (67.3)
**Prevalent cases**	N (%)	17 (32.7)
**Inclusion status**		
*Familial screen cases*	N (%)	14 (26.9)
*Index cases*	N (%)	38 (73.1)
**Hematopoietic stem cell transplant (HSCT) status**		
*Patients with allogenic HSCT*	N (%)	19 (36.5)
*Patients without allogenic HSCT*	N (%)	29 (55.8)
*Patients with gene and auto HSCT therapy*	N (%)	3 (5.8)
*Missing value*	N (%)	1 (1.9)

ALD: adrenoleukodystrophy; CCALD: childhood cerebral ALD

### CCALD natural history (objective B)

Among the 52 patients included in this study, 47 (90.4%) had at least one year of clinical history before index date and sufficient follow-up (i.e. 6 months minimum) (Fig. 1). Twenty-nine (29, 61.7%) of them did not undergo HSCT, while 18 (38.3%) had HSCT.

Median ages at ALD diagnosis (VLCFA dosage, ABCD1 mutation detection – detection of every ALD subtypes) and CCALD diagnosis (clinical description) were both 7.0 years.

Major functional disability (MFD) gathers different CCALD-related complications including loss of communication, use of a wheelchair, incontinence, blindness, use of enteral feeding (nasogastric/gastrostomy) and loss of movement (Table 2).

**Table 2:** CCALD natural history, symptomatology and survival

Characteristics		No HSCT (N = 29)	HSCT (N = 18)	Total (N = 47)
Diagnoses				
*Age at ALD diagnosis (y)*	Median (Q1-Q3)	7.0 (6.0–10.0)	6.0 (5.0–8.0)	7.0 (5.0–9.0)
*Age at CCALD diagnosis (y)*		7.0 (6.0–10.0)	7.0 (6.0–9.0)	7.0 (6.0–10.0)
*Time between diagnoses (months)*		0.0 (0.0–0.0)	1.3 (0.0–16.1)	0.0 (0.0–0.0)
**Symptoms**				
*Patients with CCALD symptoms*	N (%)	27 (93.1)	11 (61.1)	38 (80.9)
*Patients with HSCT-preventable symptoms*		27 (93.1)	3 (16.7)	30 (63.8)
*Patients without symptoms*		2 (6.9)	7 (38.9)	9 (19.1)
*Age at 1st CCALD symptom (y)*	Median (Q1-Q3)	7.0 (6.0–9.0)	7.0 (6.0–9.0)	7.0 (6.0–9.0)
**CCALD main symptoms**				
*Cognitive symptoms*	N (%)	15 (51.7)	1 (5.6)	16 (34.0)
*Motor symptoms*		8 (27.6)	0 (0.0)	8 (17.0)
*Adrenal insufficiency*		0 (0.0)	9 (50.0)	9 (19.1)
*Seizures*		1 (3.4)	0 (0.0)	1 (2.1)
*Other symptoms*		5 (17.2)	2 (11.1)	7 (14.9)
**Major Functional Disabilities (MFD)**				
*Patients with MFD*	N (%)	18 (62.1)	3 (16.7)	21 (44.7)
*Patients without MFD*		11 (31.9)	15 (83.3)	26 (55.3)
*Age at 1st MFD*	Median (Q1-Q3)	7.0 (6.0–12.0)	10.0 (10.0–21.0)	8.0 (6.0–12.0)
**Main MFDs**				
*Communication loss*	N (%)	16 (80.0)	0 (0.0)	16 (42.1)
*Use of wheelchair*		14 (48.3)	0 (0.0)	14 (29.8)
*Incontinence*		7 (24.1)	0 (0.0)	7 (14.9)
*Blindness*		10 (34.5)	3 (16.7)	13 (27.7)
*Enteral feeding*		11 (37.9)	0 (0.0)	11 (23.4)
*Movement loss*		9 (31.0)	0 (0.0)	9 (19.1)
MFD-free survival*				
*Median MFD-free survival (months)*	Median (Q1-Q3)	20.0 (3.3–84.0)	NE (NE – NE)	41.9 (20.0 – NE)
*% at 12 months after diagnosis*	% [CI95%]	51.1 [31.8 ; 67.5]	94.4 [66.6;99.2]	67.9 [52.5;79.3]
*% at 60 months after diagnosis*		31.9 [14.6;50.6]	72.2 [45.6;87.4]	48.4 [33.0;62.2]
Overall survival*				
Median age at death (y)**	Median (Q1-Q3)	10.0 (8.0–16.0)	9.0 (9.0–9.0)	10.0 (8.0–16.0)
*5-year overall survival*	% [CI95%]	66.6 [41.0;83.2]	94.4 [66.6;99.2]	80.0 [63.8;89.5]
*End of study overall survival*		30.5 [6.2;60.3]	94.4 [66.6;99.2]	65.5 [44.2;80.3]

* Survivals were assessed based on Kaplan-Meier estimates

**Only one patient with HSCT died

ALD: adrenoleukodystrophy; CCALD: childhood cerebral ALD; HSCT: allogenic stem cell transplantation; MFD: major functional disabilities

CCALD symptoms gather: cognitive symptoms, adrenal insufficiency, motor symptoms, convulsions and other symptoms

MFD gathers: loss of communication, wheelchair, incontinence, blindness, enteral feeding (nasogastric/gastrostomy) and loss of movement

HSCT only refers to allogenic transplantations

**Fig. 2:**
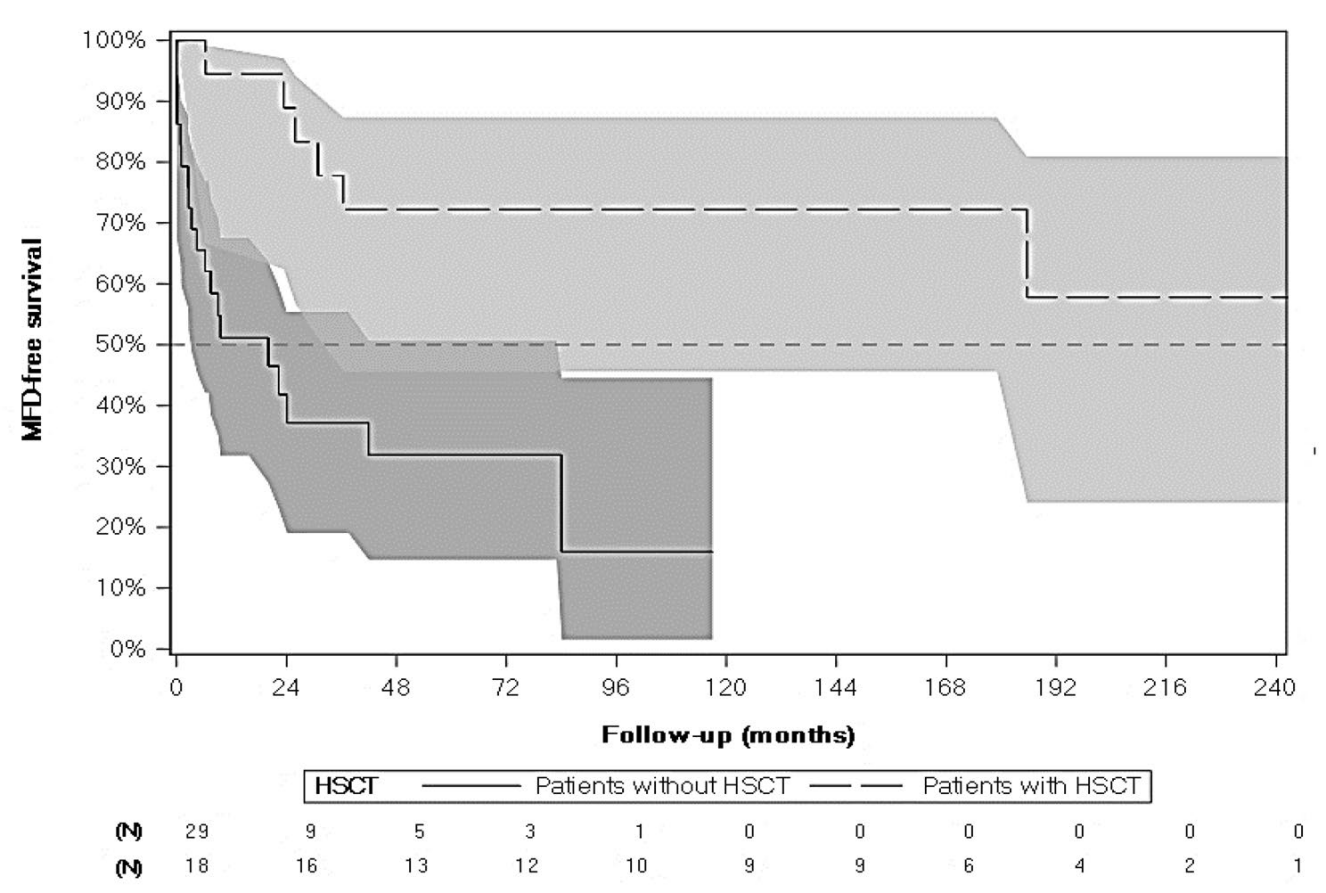
MFD-free Survival (months) from CCALD Diagnosis for Patients with and without HSCT - Kaplan-Meier (N = 47) *MFD gathers: loss of communication, use of wheelchair, incontinence, blindness, use of enteral feeding (nasogastric/gastrostomy) and loss of movement. HSCT only refers to allogenic transplantations*

Almost half of patients (21, 44.7%) presented at least one MFD, with a MFD-free survival [CI95%] of 94.4% [66.6%; 99.2%] 12 months after CCALD diagnosis decreasing to 72.2% [45.6%; 87.4%] 60 months after CCALD diagnosis. Overall crude mortality rate was 23.4% (Table 2 – Fig. 2).

#### CCALD patients without HSCT

Among the 29 patients without HSCT, median (Q1-Q3) ages at ALD and CCALD diagnoses were both 7.0 (6.0–10.0) years of age, ranging from 5 to 13. These two diagnoses were concomitant for the entirety of the cohort without HSCT.

**Fig. 3:**
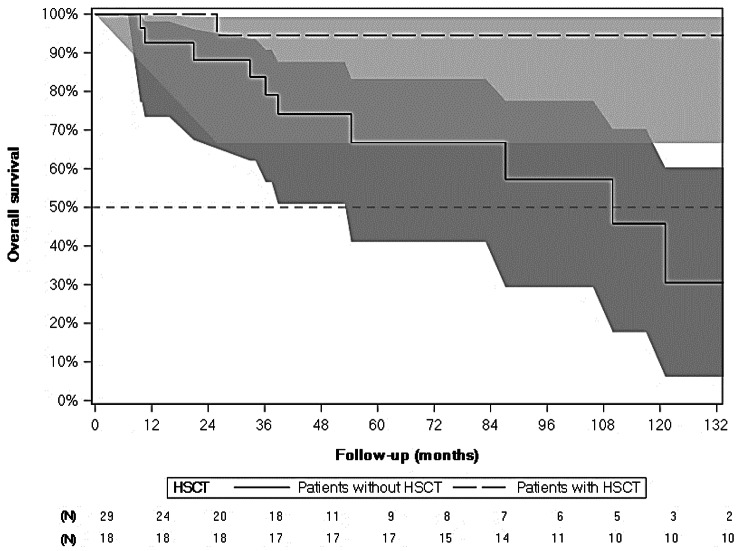
Overall Survival (months) from CCALD Diagnosis for Patients with and without HSCT - Kaplan-Meier (N = 47) *HSCT only refers to allogenic transplantations*

Crude mortality rate was 34.5%, with 10 patients dying during study period. Median age at death was 10 years, with two patients dying within the year after diagnosis. Five-year overall survival [CI95%] was 66.6% [41.0%; 83.2%], while overall survival at the end of follow-up was 30.5% [6.2%; 60.3%] based on Kaplan-Meier estimate (Table 2 – Fig. 3).

Considering CCALD symptoms, the majority of them (27, 93.1%) presented at least one, with a median age at onset of 7.0 years, ranging from 3 to 12. Most of them (25, 92.6%) had symptoms before CCALD diagnosis, with a median period of 6.9 months between symptom occurrence and CCALD diagnosis. Cognitive and behavioral symptoms were the most frequent first CCALD symptoms to occur, encountered among half of patients (15, 51.7%). Motor symptoms were second, occurring in 27.6% (n = 8) of cases, while other symptoms were encountered in 17.2% (n = 5) of patients. Only two (6.9%) patients did not present any symptom (Table 2).

Eighteen (18, 62.1%) patients without HSCT presented at least one MFD, occurring at 7 years of age in median, either before or within the year after CCALD diagnosis. A median of 5 MFDs was encountered among them. Communication loss was the most frequent MFD, with 16 patients (34.0%). Need for a wheelchair and enteral feeding came second and third, with 14 (29.8%) and 11 (23.4%) patients, respectively. Communication loss was also the MFD with the earliest onset, with a median age at onset of 7.0 years, while movement loss and incontinence were of the latest onset with 13 years of age in median (Table 2). Median MFD-free survival was 20 (3.3–84.0) months. It was 51.1% [31.8%; 67.5%] 12 months after CCALD diagnosis and decreased to 31.9% [14.6%; 50.6%] 60 months after CCALD diagnosis (Table 2 – Fig. 2).

#### CCALD patients with HSCT

Among the 18 patients who underwent HSCT, median (Q1-Q3) age at ALD diagnosis was 6.0 (5.0–8.0) years and was 7.0 (6.0–9.0) years at CCALD diagnosis, all comprised between 0.5 and 13 years of age. Unlike patients without HSCT, ALD and CCALD diagnoses were not concomitant, with a median (Q1-Q3) time between them of 1.3 (0.0–16.1)months.

Crude mortality rate was 5.6%, as only one patient with HSCT died during study period at the age of 9, 26 months after CCALD diagnosis. Five-year overall survival [CI95%] was 94.4% [66.6%; 99.2%], which remained stable until the end of the study (Table 2 – Fig. 3).

Only 2 patients with HSCT (11.1%) presented HSCT-preventable CCALD symptoms (i.e. excluding adrenal insufficiency – encountered in 9 patients – which is not impacted by HSCT). Symptoms encountered were cognitive and behavioral symptoms. The remaining 16 (88.9%) patients did not present symptoms (Table 2).

Similarly to symptoms, only 3 (16.7%) patients with HSCT presented a MFD, all being blindness, occurring at 10 years of age in median, 3 years after CCALD diagnosis. Median MFD-free survival was not calculable as less than 50% of patients with HSCT either presented a MFD, needed a second HSCT or died during study period. MFD-free survival was 94.4% [66.6%;99.2%] 12 months after CCALD diagnosis and decreased to 72.2% [45.6%;87.4%] after 60 months after CCALD diagnosis (Table 2 – Fig. 2).

### HSCT use according to diagnosis settings (objective C)

Among the 52 patients included in this study, 48 (92.3%) had a sufficient follow-up (i.e. 6 months minimum) after CCALD diagnosis (Fig. 1).

Of them, 19 (39.6%) patients had an allograft after CCALD diagnosis; 10 being screened cases after family analysis, and 9 being index cases. Proportions of HSCT among screened and index cases were 83.3% and 25.0%, respectively. Two (2) patients received at least one subsequent HSCT during study period.

Median (Q1 – Q3) age at HSCT was 7.0 (6.0–10.0) and 8.0 (7.0–10.0) among screened and index cases, respectively. Time between CCALD diagnosis and HSCT was 4.9 (1.4–9.2) months in median among index cases. It was doubled among screened cases with 10.3 (3.0–51.4) months (Table 3).

**Table 3:** Allogenic Stem Cell Transplantation (HSCT) use

Characteristics		Familial screen cases (N = 10)	Index cases (N = 9)	Total (N = 19)
Allo-HSCT				
*Age at 1st HSCT (y)*	median (Q1-Q3)	7.0 (6.0–10.0)	8.0 (7.0–10.0)	7.0 (6.0–10.0)
*Time between diagnosis and HSCT (months)*		10.3 (3.0–51.4)	4.9 (1.4–9.2)	4.9 (1.4–19.9)

Due to an important proportion of missing data concerning HSCT conditions among patients with HSCT (12, 63.2%), HSCT complications could not be presented in this study.

### CCALD healthcare resource use (objective D)

Among the 52 patients included in this study, 48 (92.3%) had a sufficient follow-up period (i.e. 6 months minimum) after CCALD diagnosis and were followed-up until end of study or death, with a median (Q1-Q3) duration of 5.9 (2.3–9.9) years. Twenty-nine (29, 60.4%) of them were patients without HSCT, while the 19 (39.6%) remaining patients underwent HSCT (Fig. 1).

#### CCALD patients without HSCT

Considering inpatient healthcare resources, almost every patient without HSCT (93.1%) were hospitalized at least once during study period, with an annual median (Q1 – Q3) of 5.4 (1.1–8.0) hospitalizations, accounting for 23.8 (2.8–121.3) days, each year. Twenty-five (25, 86.2%) patients had at least one ER visit, with an annual median of 1.2 ER visits. Among patients without HSCT who had an ICU stay (< 11), annual length of stay in ICU was 4.5 (2.1–6.0) days in median.

Regarding outpatient healthcare resources, patients without HSCT had a median of 2.9 (0.9–6.3) medical visits per year, mostly represented by hospital specialist visits. Twenty-five patients (86.2%) had at least one paramedical care during study period (including nursing care, physiotherapy, podology, speech therapy, orthoptist), almost exclusively represented by nursing care and physiotherapy. Median annualized number of days with any paramedical care, and for nursing care and physiotherapy were 12.7 (1.7–107.9), 1.5 (0.0–10.3) and 1.5 (0.0–97.9), respectively (Table 4).

**Table 4:** Healthcare Resource Use

Characteristics		No HSCT (N = 29)	HSCT (N = 19)	Total (N = 48)
Hospitalizations				
*At least one hospitalization*	N (%)	27 (93.1)	18 (94.7)	45 (93.8)
*Annualized number of hospitalizations*	median (Q1-Q3)	5.4 (1.1–8.0)	1.6 (0.5–3.7)	2.9 (0.6–6.2)
*Annualized cumulative length of stay (d)*	median (Q1-Q3)	21.1 (2.8–89.6)	6.2 (1.0–13.6)	10.9 (1.4 − 24.1)
**Emergency room (ER)**				
*At least one ER visit*	N (%)	25 (86.2)	14 (73.7)	39 (81.3)
*Annualized number of ER visits*	median (Q1-Q3)	1.2 (0.3–1.9)	0.4 (0.0–1.0)	0.9 (0.2–1.6)
**Intensive care unit (ICU)**				
*At least one ICU stay*	N (%)	< 11	< 11	18 (40.0)
*Annualized cumulative length of stay (d)*	median (Q1-Q3)	4.5 (2.1–6.0)	7.4 (5.0–9.6)	5.9 (2.1–9.6)
**Magnetic Resonance Imaging**				
*At least one brain MRI*	N (%)	21 (72.4)	14 (73.7)	35 (72.9)
*Overall Number of MRI*	median (Q1-Q3)	2.0 (0.0–6.0)	3.0 (0.0–11.0)	2.0 (0.0–6.0)
Medical visits*				
*At least one medical visit*	N (%)	27 (93.1)	17 (89.5)	44 (91.7)
*Annualized number of medical visits*	median (Q1-Q3)	2.9 (0.9–6.3)	3.9 (1.2–6.5)	3.2 (1.0–6.4)
Paramedical care**				
*At least one paramedical care*	N (%)	25 (86.2)	17 (89.5)	42 (87.5)
*Annualized number of paramedical care*	median (Q1-Q3)	12.7 (1.7–107.9)	5.9 (0.7–11.1)	6.1 (1.3–83.4)
**Medical devices**				
*Medical bed*	N (%)	14 (48.3)	< 11	NE
*Wheelchair*	N (%)	12 (41.4)	< 11	NE
*Orthesis*	N (%)	< 11	< 11	NE
**Medical transportations**				
*At least one medical transport*	N (%)	19 (65.5)	< 11	NE
*Annualized number of medical transport*	median (Q1-Q3)	1.1 (0.0–11.2)	0.0 (0.0–0.5)	0.3 (0.0–5.1)

*Medical visits gather: rehabilitation specialist, neurologist, urologist, pediatrician, general practitioner, hospital specialist

**Paramedical care gathers: nursing care, physiotherapist, podologist, speech therapist, orthoptist

HSCT only refers to allogenic transplantations

#### CCALD patients with HSCT

Considering inpatient healthcare resources, almost every patient with HSCT (94.7%) were hospitalized at least once during study period, with an annual median (Q1 – Q3) of 1.6 (0.5–3.7) hospitalizations, accounting for 6.2 (1.0–13.6) days, each year. Fourteen (14, 73.7%) patients had at least one ER visit, with an annual median of 0.4 ER visits. Among patients with HSCT who had an ICU stay (< 11), annual length of stay in ICU was 7.4 (5.0–9.6) days in median.

Regarding outpatient healthcare resources, patients with HSCT had a median of 3.9 (1.2–6.5) medical visits per year, mostly represented by hospital specialist visits. Seventeen patients (89.5%) had at least one paramedical care during study period, almost exclusively represented by nursing care and physiotherapy. Median annualized number of days with paramedical care was 5.9 (0.7–11.1), 0.9 (0.2–2.0) and 1.1 (0.0–5.8) overall, and regarding nursing care and physiotherapy, respectively (Table 4).

### Costs associated with CCALD management (objective D)

**Fig. 4:**
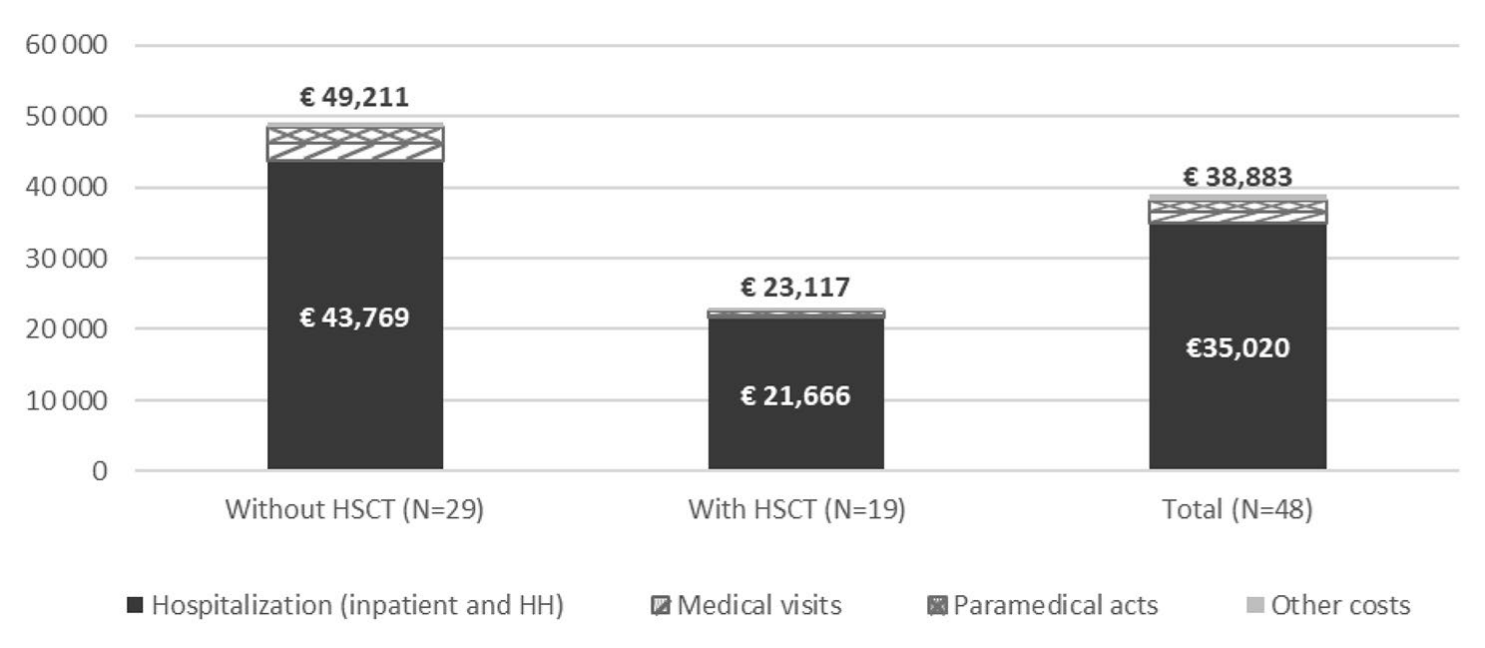
Mean annualized Costs (€) for patients with and without HSCT during Study Period (N = 48) *HH: home hospitalization* *Other costs gather medical devices, laboratory tests, procedures, medications and transportations* *HSCT only refers to allogenic transplantations*

Annualized costs related to healthcare resources used during study period were 38,883€ (45,458€), with more than 90% (35,020€) for hospitalizations (including ER visits). Overall, paramedical care, transports and medications came in second, third and fourth, with mean annualized costs of 1,672€, 1,493€ and 267€, respectively. Medical procedures and laboratory tests were last with less than 100€ per year (Fig. 4).

#### CCALD patients without HSCT

Among the 29 patients without HSCT, mean (SD) annualized related costs were 49,211€ (52,623€), with 88% (43,769€) for hospitalizations. Medical transports were the second most important item of expenditure with 2,341€ (7,637€) (Fig. 4).

#### CCALD patients with HSCT

Among the 19 patients with allograft, mean (SD) annualized costs were 23,117€ (25,573€), with more than 93% (21,666€) for hospitalizations. These costs included the allograft stay fee, being more than 300,000€ by itself. Paramedical care was the second most important item of expenditure with 686€ (1,288€) (Fig. 4).

#### CCALD patients with HSCT – HSCT cost evolution

Among the patients who underwent HSCT between 2009 and 2018 (i.e. period with SNDS data available), seven presented sufficient historical period and data granularity to assess cost evolution around HSCT and up to end of study period. During the 6 months prior to HSCT, mean overall costs were 15,096€. During the first year following HSCT (HSCT included), costs peaked at 309,101€, with hospitalization accounting for 306,241€. Then a decrease was observed during the second and subsequent years post-HSCT, with mean costs of 9,289€ during the second year, and an annualized mean cost of 3,020€ after (Fig. 4).

## Discussion

### Study population

To the best of our knowledge, this study is the first one including this type of linkage in CCALD literature, allowing availability of both national database exhaustiveness and specific registry granularity. Based on literature, X-linked ALD incidence in France is estimated around 1/17,000 births, of whom 35–40% might develop CCALD, leading to an expected annual incidence between 5 and 6 young boys in France [1–4]. In current study, median annual incidence was 4, and reached expected incidence only twice. Apart from a limited underestimation due to partial linkage of patients (93%), the main cause for underestimation was expected to be a potential recruitment bias, highlighted by the rarity of the disease and its difficult diagnosis. As only patients referred to reference centers are included in LEUKOFRANCE registry, patients with very late diagnosis who already present severe CCALD symptoms or MFDs could not be referred to the registry, hence not captured in this study.

### Patients’ characteristics and symptomatology

More than half of patients were diagnosed between 5 and 8 years of age, with a median age of 7. Mallack & al meta-analysis including 107 studies from 1970 to 2019, showed similar results, in line with CCALD clinical manifestations appearing between 3 and 12 years of age, usually [5, 29–31]. However, the absence of specific symptoms, notably for patients without family history, makes diagnosis more difficult and requiring for trained professionals. As HSCT is effective among youngest patients, neonatal diagnosis test would be a good option, even it is not implemented in France. In 2018, Bessey & al estimated the potential economic impact of implementing X-linked ALD within routine newborn screening program in United Kingdom. CCALD new-born screening has been shown as dominant in almost every tested scenario, when compared to no screening [32]. In current cohort, almost 25% of patients were screened, and more than 80% of them were transplanted and did not present CCALD symptoms during study period. In addition to newborn screening, a familial screening could allow an early detection of mutation carriers, targeted genetic counselling, and precocious therapies among patient’s family when needed. Several studies and case-reports showed the importance of familial screening. Hetman & al reported a CCALD diagnosis in the oldest boy of a brotherhood of 3 at an advanced stage. It led to a screening on his first younger brother still asymptomatic at age 7, who could undergo HSCT, and on the youngest one, at birth [33]. When analyzing time between ALD and CCALD diagnoses, it appeared that diagnoses were almost always concomitant among index cases, which is explained by the fact that index cases are mostly diagnosed after CCALD symptoms occurrence, then labelled as ALD. As HSCT is almost ineffective on adrenal insufficiency, more than 80% of patients with HSCT did not present any HSCT-preventable CCALD symptoms (i.e., excluding adrenal insufficiency). This result highlights the importance of HSCT in CCALD management, since almost no treatment can be proposed to patients with neurological defects, while oral therapies are available to compensate adrenal glands [34–36]. Only 3 patients with HSCT presented a MFD (cortical blindness). This finding shows the importance of early HSCT, as loss of eyesight in CCALD is progressive but irreversible, hence HSCT cannot repair present damages and is known to be fully effective after 6 to 9 months [37, 38]. In patients with HSCT, MFD-free survival was more than 70% 5 years after CCALD diagnosis. Half of patients without HSCT presented either MFD or death 20 months after CCALD diagnosis. In Kühl & al single center case study MFD-free survival was similar, with 64% after 10 years [31]. Five (5)-year overall survival was estimated at 66.6% for patients without HSCT, slightly higher than in the literature, which is around 55% [8, 39]. CCALD known high variability of both clinical presentation and management, can partly explain this discrepancy. Another hypothesis can be the presence of a recruitment bias, as described above, since the totality of patients included in our study come from LEUKOFRANCE registry. Among patients with HSCT, 5-year overall survival was estimated at 93%, in line with data from Raymond & al multicenter study [8].

### CCALD management

One identified study limitation was the lack of information regarding HSCT donor. In Europe, EBMT report showed that less than 20% of CCALD patients who underwent allogenic HSCT did not have matched sibling donor with complete histocompatibility (HLA 10/10), leaving 80% with Matched Unrelated Donors or MisMatched Unrelated Donor [40]. In France, in order to maximize satisfying engraftment, HSCT are almost exclusively reserved to patients with complete histocompatibility. This could notably explain the longer period between diagnosis and HSCT among screened cases, as the early diagnosis decreases the urgency of the transplantation and allows a longer time for graft research. It is of note that GVHD and other complications such as infections, have been shown to have non-negligible impact on allografted patient evolution in literature. Raymond estimated that 45% and 21% of patients presented acute or chronic GVHD, respectively, with infections GVHD (either acute or chronic) recorded as cause of death among several patients [8]. Also, SNDS database does not provide information on the reasons of healthcare resources used, notably HSCT. Hence it is possible that some patients could have received HSCT for another indication, notably malignancies or myelodysplasia. However, in addition to accounting for a small proportion of the transplanted patients, the transplant would also have had a beneficial impact on CCALD, limiting the impact of this bias.

### CCALD economic burden

This study is the first to directly assess HCRU and related costs through real-life data in France.

Patients who had HSCT spent less days per year hospitalized. This can be partly explained by the fact that most patients who did undergo HSCT might only encounter complications related to HSCT, and not to CCALD evolution. Despite the relative importance of HSCT-related complications, incidence of these events remains highly lower than those linked to CCALD which are part of natural disease expansion. Regardless of time period and HSCT status, a vast majority (> 90%) of CCALD economic burden seemed attributable to hospital care. As expected, peak cost of care was reached with HSCT. Despite the costs of stem cells themselves and infusion act, a non-negligible proportion of HSCT-related cost comes from a potential prolonged stay in hematological ICU, due to patient’s aplasia. Also, HSCT remains highly effective for long-term HCRU, and related cost decrease once discharged from HSCT stay, with mean annualized cost among study period almost divided by 2 when comparing patients with and without HSCT. Annualized costs remain a robust approach to estimate CCALD burden. For instance, when mean annual costs are multiplied by follow-up period, total CCALD related costs was estimated around 138,000€ and 273,000€ respectively for patients with and without HSCT, respectively, highlighting a consequent burden though life. When analyzing cost evolution with time and disease progression, two completely different patterns are expected between patients with HSCT and those without HSCT. As HSCT is usually performed within months after CCALD diagnosis, a peak could be reached with HSCT, followed by a drastic decrease and a low plateau. In the other hand, as disease progresses among patients without HSCT, costs might be quite low for the first months unlike with HSCT but will progressively and inexorably increase with symptoms and MFD onset. This study only estimated direct medical costs (e.g., inpatient related costs, transportations, laboratory test etc…). However, CCALD also has an important impact on non-medical, indirect, and intangible costs. Bessey et al. study included part of these indirect costs as social care costs, estimating a burden of 6.44 million £ for 18.3 X-ALD cases (9.7 CCALD cases), potentially leading to more than 360,000€ per child [32], overall. Caregivers are expected to take sick child leaves, or even fulltime care periods, strongly impacting their own productivity and quality of life. Also, among direct medical cost which were not available in SNDS data, some might be of significant importance for caregivers. Out of pocket expenses for partially reimbursed medications or medical devices, specialist fee overruns, use of alternative medicines, house adaptation for bedridden patients are several examples of other cost inputs not included in SNDS database, and which are at caregiver’s charge or covered by associations.

Finally, SNDS database does not include information on quality of life. However, Bessey et al. transposed ALD clinical score (ALD-DRS) into quality-of-life score (EQ-5D). For the lowest levels (ALD-DRS I / ALD-DRS II), corresponding to mild neuro-sensorial and behavioral impairments, the related EQ-5D scores were around 0.6, with 1.0 being the best health state possible. Non-transplanted CCALD patients are expected to present the highest scores, being ALD-DRS III and IV, the related EQ-5D were 0.11 and 0.031 respectively. It seems acceptable to assess that transplanted patients, if treated early enough, would present scores equal or higher to ALD-DRS I, highlighting the importance of HSCT on every outcomes of interest, either clinical, economic, or of quality of life [32].

## Conclusion

X-linked ALD is a rare metabolic and neurodegenerative genetic disease, causing irreversible damages on peripheral nervous system and adrenal glands. Birth incidence of X-linked ALD is estimated around 1/17,000, of whom 40% will develop early symptoms, called Childhood Cerebral ALD (CCALD), the most devastating ALD form. This study estimated the number of CCALD patients in France, their clinical characteristics, therapeutic management and medium- and long-term evolution, through a follow-up analysis of 6 years in median. With 4 incident CCALD patients per year in France, and despite a potential underestimation due to patients’ non-exhaustive referral to LEUKOFRANCE registry, CCALD rarity has been highlighted. Until recently, HSCT was the only treatment able to stop CCALD progression but had to be performed as early as possible to prevent irreversible damages. Only a small amount of CCALD patients, less than 40% in our study, is eligible to HSCT. However, when performed, HSCT brings drastic improvement in terms of overall clinical presentation and overall lifespan. In addition, CCALD economic burden seemed highly linked to HSCT status, with a three-time increase of annual hospital length of stay (6.2 days vs. 21.1 days) and a two time increase of overall annual costs (23,117€ vs. 49,211€).

HSCT in CCALD disease management has been shown as primordial when performed early enough to prevent symptoms and MFDs. The necessity of a precocious management highlights the potential benefits of including an expanded screening program among newborns, coupled with family screenings when a mutation is detected.

To the best of our knowledge, this is the most up to date study analyzing CCALD epidemiology, clinical and economic burden in France. It is also the first one to link complete reimbursement database (SNIIRAM) to a clinical ALD specific registry (LEUKOFRANCE) in France. It allowed both precise clinical burden and disease evolution assessment through clinical data, as well as good estimates for CCALD economic burden among patients who did and did not undergo HSCT.

## Data Availability

No additional data available.

## List of Abbreviations

ALD: Adrenoleukodystrophy
ALDP: Adrenoleukodystrophy Protein
(Allo)-HSCT (allogeneic): Hematopoietic Stem Cell Transplantation
AMN: Adrenomyeloneuropathy
ATC: Anatomical, Therapeutic and Chemical classification
CCALD: Childhood Cerebral ALD
CCAM: Medical procedures classification (*Classification Commune des Actes Médicaux*)
CIP: French drug package code (*Code Identifiant de Présentation)*
ER: Emergency Room
GVHD: Graft Versus Host Disease
ICD-10: International Classification of Diseases – 10th revision
ICU: Intensive Care Unit
INSEE: National institute for statistics (*Institut National de la Statistique et des Etudes Economiques*)
LPP: Medical devices list (*Liste des Produits et Prestations*)
MFD: Major Functional Disabilities
MRI: Magnetic Resonance Imaging
NABM: Laboratory test classification (*Nomenclature des Actes de Biologie Médicale*)
NFS: Neurological Functional Score
Q1-Q3: Quartiles
SNDS: French National Health Data System (*Système National des données de Santé*)
SD: Standard Deviation
